# Traffic Measurement on Multiple Drive Lanes with Wireless Ultrasonic Sensors

**DOI:** 10.3390/s141222891

**Published:** 2014-12-02

**Authors:** Soobin Jeon, Eil Kwon, Inbum Jung

**Affiliations:** 1 Department of Computer Information and Communication Engineering, Kangwon National University, Chuncheon, 200-701, Korea; E-Mail: sbjeon@kangwon.ac.kr; 2 Civil Engineering, University of Minnesota Duluth, Duluth, MN 55812, USA; E-Mail: eilkwon@d.umn.edu

**Keywords:** ITS, ultrasonic sensor, lateral scanning, vehicle detection, sensor network

## Abstract

An automated traffic measuring system for use on multiple drive lanes is proposed in this paper. This system, which uses ultrasonic sensors and a lateral scanning method, is suitable for use on real traffic roads. The proposed system can be easily established and maintained in various roadway environments. In addition, the system can be adjusted to measure traffic volumes according to the size and number of drive lanes. This paper describes the results of an experiment that the lateral scanning method can be easily applied to real traffic roads and provide a low error rate and real-time responses. This system can play an important role in accurately measuring traffic volumes as part of an intelligent transportation system.

## 1. Introduction

The development of intelligent transportation systems has had a great influence on many aspects of road transportation systems. Traffic measurement technology in particular, conducted using various types of detection devices, has had an effect on the analysis of traffic flow. Loop detectors, which are installed beneath road surfaces, have been mainly used in the past for traffic measurement. However, loop detectors often break as a result of damage from vehicles that pass over them, and they have high maintenance costs [1].

Ultrasonic sensors are frequently used as vehicle detection devices because they are cheaper and more accurate than other types of devices. Most ultrasonic sensors detect vehicles by measuring from top to bottom or from side to side diagonally. However, these approaches require that a detector be installed for each lane because each detector measures only one lane on a road. Furthermore, ultrasonic sensors require considerable infrastructure on a road [2].

In this paper, we propose a lateral scanning method for measuring the traffic on multiple lanes of a road using a wireless sensor network and ultrasonic sensors. The proposed system detects and classifies vehicles from the side of a road. Whenever an ultrasonic sensor detects the passage of a vehicle on the road, the system measures the distance to the corresponding vehicle based on its lane location. Vehicle detection and lane classification can be performed using the data on the distance to the vehicle and the time required for the vehicle to pass through the detection range of the ultrasonic sensor. The detection algorithm consists of three parts. The first part calculates thresholds, which are points at which vehicles are detected in each lane. The second part filters out unnecessary data, such as noise from the natural environment. The third part determines the locations of vehicles in multiple lanes and calculates the traffic volume, based on the filtered data and the calculated thresholds.

To test our proposed system, we developed a new device called a wireless ultrasonic sensor mote (WUSM), which is small and easy to install and can be adapted for use in a variety of real road environments. The accuracy and efficiency of the proposed system for vehicle detection and classification using WUSMs were evaluated. The results of detailed experiments show that the proposed system be easily established and maintained in various roadway environments and can measure traffic volumes by vehicle size and the number of drive lanes.

The remainder of this paper is structured as follows: first an overview of existing ultrasonic sensor technology is provided. The proposed vehicle detection algorithm and system module are then described. The experiments conducted to evaluate the performance of the proposed system and the results of those experiments are summarized. Finally, conclusions drawn from the results and plans for future work are presented.

## 2. Overview of Ultrasonic Detection Systems

The collection of vehicle information, including the traffic flow rate and the presence, speed, and location of vehicles, is the most basic requirement for an intelligent transportation systems (ITS). Surveillance technologies can be classified as intrusive or non-intrusive technologies. Intrusive traffic sensors are installed within or across a pavement. Non-intrusive sensors can be installed above or on the sides of roads, with minimum disruption to traffic flow.

Vehicle sensing technologies include inductive loops, which are most commonly used in roads, pneumatic road tubes, piezoelectric cables, and weigh-in-motion systems. In general, these technologies require installation directly into or onto pavements by insertion through holes or tunneling under the surface, and they are most often used to accurately detect the presence of vehicles on the pavement. However, intrusive detection technologies have drawbacks, including disruption of traffic for installation and repair, failures induced by poor road conditions, system reinstallation necessitated by road repairs or resurfacing, and high maintenance costs.

Non-intrusive technologies include microwave radar, infrared (IR)-based systems, video image processing (VIP), and ultrasonic detectors. These devices are usually set up on the roadside or overhead position. Non-intrusive technologies therefore offer advantages that include low installation costs, ease of access for maintenance, and installation and repair without disruption of traffic. However, non-intrusive detectors also have drawbacks, include their relatively low accuracy compared to that of intrusive detectors.

The main advantage of microwave radar is that the system performance is not affected by weather changes. However, microwave radar cannot detect motionless vehicles without the aid of an auxiliary device. Infrared systems are capable of transmitting multiple beams for purposes of multi-zone detection using a single detector unit, but their performance is greatly affected by the environment: sunlight can confuse the signals, and IR energy can be absorbed and scattered by atmospheric particulates, fog, rain, and snow. The performance of VIP systems is also greatly affected by inclement weather: false detections can be caused by vehicle shadows falling on adjacent lanes, and camera vibrations can be caused by strong winds. Other disadvantages of VIP systems include the height at which the cameras must be mounted above the road (up to 60 feet high), the relatively high installation and equipment costs, and the fact that the system is only cost effective if many detection zones are required within the field of view of the camera [1,3,4].

The system proposed in this paper employs a detection algorithm based on ultrasonic sensors. “Ultrasonic” refers to high-frequency sound waves that are beyond a human's audible range. Waves with frequencies between 25 and 50 kHz are commonly used. The principal mechanism is similar to that of microwave radar. Sound pulses are transmitted, the reflected pulses are received, and the distance from the receiver to the road or a vehicle surface is calculated from the wave travel time. The performance of ultrasonic sensors is much better than that of other types of pulse devices. Ultrasonic detection systems can detect vehicles in multiple zones and measure their speeds, and they are much cheaper than intrusive systems. Also, they have disadvantages that its performance is affected by temperature change and air turbulence. However, some modern models, such as those used in our proposed system, have built-in temperature compensation.

**Figure 1.**
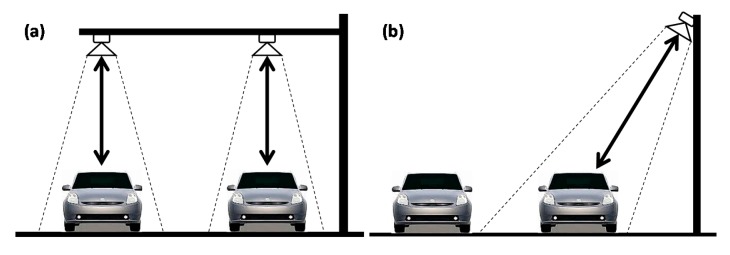
Ultrasonic sensor detection methods. (**a**) Overhead mount and (**b**) side top mount.

Typically, an ultrasonic sensor transmits a sound pulse from above the road and measures the reflected pulses from the vehicle or ground, as shown in Figure 1a. Once the default distance from the detector to the ground is set, if a vehicle passes through the detection range of the ultrasonic sensor, the distance value changes depending on the vehicle's size, and the detection system detects the presence of the vehicle based on the distance data received. The vehicle detection accuracy achieved using this method is approximately 99.5% for each ultrasonic sensor installed on each lane. This method has the advantage of being able to detect vehicles accurately, but it has the disadvantage that a sensor must be installed on each lane. There is also a risk of damage to the sensors, depending on the heights of the vehicles [5–7].

The method represented in Figure 1b detects diagonal distances to vehicles from roadside-mounted sensors. When the sensors receive waves reflected from the edges of vehicles, they detect the vehicles' presence and speeds based on the distances from the vehicles. The accuracy of this method, which is approximately 93%–95%, is lower than the previously described method by which vehicles are detected by sensors mounted above the road, because wave signals reflected only from the edges of vehicles are weak. This method also has the disadvantage that a sensor must be installed for each lane [8,9].

## 3. Development of a Lateral Scanning-Based Ultrasonic Sensing System

### 3.1. Architecture

The ultrasonic sensor system in current use can only detect vehicles in one lane with one device, and it is not easy to install many devices on the side of a road because the devices are large and expensive. To address the problem of only being able to detect vehicles in one lane with one device, we propose a new detection system that also offers the advantages of easy mobility via a wireless sensor network and miniaturization via specially fabricated measuring devices. In addition, the new system can detect vehicles in multiple lanes with just one device.

**Figure 2.**
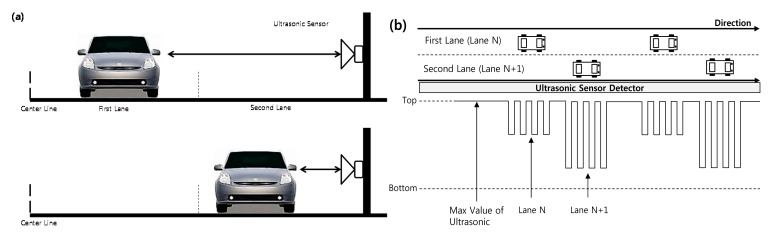
(**a**) Side-fire configuration for vehicle detection and (**b**) detection data from wireless ultrasonic sensor.

To detect vehicles using ultrasonic sensors, we use a side-fire configuration with ultrasonic sensors positioned diagonally on the side of the road. Figure 2a shows the side-fire configuration for vehicle detection proposed in this paper, including the location of first lane and second lane from the center line and the location of ultrasonic sensor. The upper drawing in Figure 2a shows a vehicle in the first lane, and the lower drawing shows a vehicle in the second lane.

When an ultrasonic wave is generated, if the distance traveled by the carrier wave equals the maximum value of the sensor distance, it means there is no vehicle in the detection range. If the sensor receives carrier wave data indicating a distance less than the maximum value, it means that a vehicle is present in the lane.

Figure 2b represents schematically how measurement data received by the ultrasonic sensor are returned from vehicles on the road along a time line. In Figure 2b, the top sign of the measurement data denotes the maximum distance from the ultrasonic sensor, and the bottom sign denotes the minimum distance. Measurements that equal the maximum value indicate that there are no vehicles in the lanes. As vehicles pass the sensor zone of the ultrasonic sensor, the distance between each vehicle and the ultrasonic sensor affects the measurement data. Based on the measured lengths, the proposed system detects the locations of vehicle in multiple lanes. In Figure 2b, we see lengths labeled as corresponding lane N and lane N+1. These represent distances to the vehicles moving in lane N and lane N+1. Three types of distances are represented: the maximum value for no vehicle, the distance to a vehicle in lane N, and the distance to a vehicle in lane N+1. Based on these measurements, the proposed system can accurately detect vehicles in each lane.

### 3.2. Vehicle Detection Algorithm

The detection algorithm is composed of three parts. First, the “Calculate Threshold” part calculates thresholds, which are points at which vehicles are detected in each lane. Second, the “Noise filtering” part eliminates unnecessary data. Third, the “Vehicle & Lane Detection” part determines the locations of vehicles in multiple lanes and calculates the traffic volume.

#### 3.2.1. Calculate Threshold

As shown in Figure 2b, when a vehicle passes the sensor zone, the ultrasonic sensor collects the carrier wave, which provides data on the distance to the vehicle. These data are used to detect the vehicles and to distinguish each lane. The distances change depending on the distances of the vehicles from the sensor. However, because there is no standard to assess the correctness of the data, it is difficult to determine the location of each vehicle.

**Figure 3.**
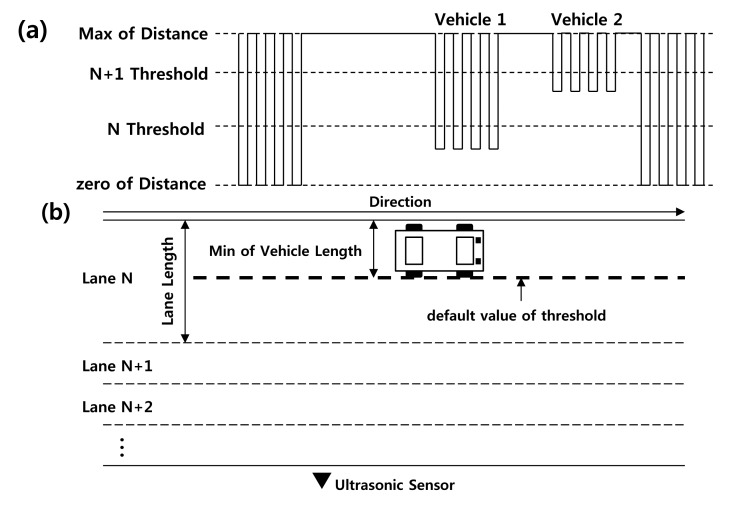
(**a**) Classification of threshold and (**b**) determination of default threshold.

To solve this problem, we define thresholds that represent the minimum boundary values for establishing the presence of vehicle in each lane. Figure 3a shows the patterns of distance data when Vehicle 1 and Vehicle 2 are moving in Lane N and Lane N+1. The boundary of Lane N is determined from the N threshold to 0 (zero distance). The boundary of Lane N+1 is determined from the N+1 threshold to the N threshold or max of distance. The max of distance is set according to number of lanes. For example, if the distance data for Vehicle 1 cross the threshold boundary of Lane N, it means that Vehicle 1 is moving in Lane N. Similarly, the distance data for Vehicle 2 indicate that Vehicle 2 is moving in Lane N+1.

The threshold boundaries are related to the widths of the lanes. The widths of the lanes for various types of roads should be defined according to the Department of Transportation in each country. The default value of the threshold (*DVT*) for a lane is determined based on these widths. On the other hand, the position of a sensor may change depending on the road status or environment. If the theoretical width of a lane is used without correction in determining the *DVT*, many errors may occur in vehicle detection and classification.

To address this problem, we established the *DVT* on the basis of both the minimum values of the widths of vehicles and the lane width data. The minimum values of the widths of vehicles are based on statistical data [10] that were examined for most vehicles on the road. If a *DVT* is set up to this value, it is possible to detect all vehicles on the road. Thus, assuming that *N* is Number of Lane, *WL* is the width of the lane and *MWV* is the minimum value of the width of the vehicle as shown in Figure 3b, the *DVTs* for each lane were defined as follows:

(1) $$DVT_{n}=\sum_{k=1}^{N}WL\times k-MWV$$

Even if an initial *DVT* were determined from Equation (1), it may differ depending on the actual road environment because it is a theoretical value based on statistical vehicle data and the standard lane width data. In this study, to reduce errors between theoretical data and actual road environments, we used corrected values of the thresholds (*CVT*s), with the corrections based on real field data obtained on actual roads. To calculate the *CVT*, an actual road vehicle is measured by threshold calculated at Equation (1). For example, if the first vehicle passes through the sensor zone and the location of the vehicle is in the *DVT*_1_ (lane 1) boundary, the distance from the first vehicle to the sensor is *CVT*_1_ value. The *CVT*s were calculated based on measurements of 500–1000 vehicles (*NoV*) using *DVT* method on real roads. Equation (2) shows how the final threshold (*FT*) can be calculated from the lowest value of the difference between the *DVT* and the *CVT*:

(2) $$FT_{n}=MIN\left[\left(DVT_{n}-CVT_{n,k}\right),\left(DVT_{n}-CVT_{n,k+1}\right),\cdots \left(DVT_{n}-CVT_{n,k+NoV}\right)\right]$$

##### 3.2.2. Noise Filtering

The ultrasonic sensor typically receives noise data that can lead to errors in the vehicle detection procedure. Noise data usually are generated as a vehicle approaches the sensor's angle or departs from it. When the vehicle departs from the sensor's angle, the sensor receives a small amount of noise because the surface area of the vehicle reflected by the carrier is also small [11,12].

**Figure 4.**
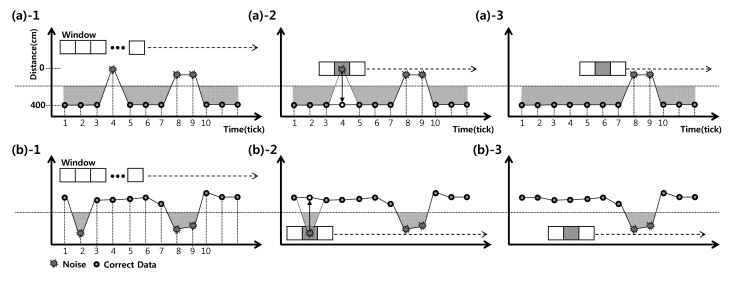
Noise Filtering. (**a**) Noise type-1 and (**b**) noise type-2.

For example, Figure 4 shows two cases of noise data received by an ultrasonic sensor. In the first case, only one vehicle passes through the sensor zone, as shown in Figure 4a-1. However, two instances of noise occur at time 4 and time 8–9. These noises suggest that three vehicles passed through the sensor zone rather than one vehicle. Figure 4b-1 shows a second case noise. There are two instances of noise at time 2 and time 8–9 that suggest that two vehicles passed through the sensor zone rather than no vehicles. Even if there are no vehicles in the sensor zone, noise can create errors in vehicle detection.

The system proposed in this paper filters out noise to prevent it causing errors in detecting vehicles. To remove noise, mask windows are first installed on the noise data, as shown in Figure 4a-2,b-2. Next, because the window is applied to the data from *i* to *i* + *k*, the trend in the data is determined based on the threshold. Assuming that *s* is the starting point of window, *k* is the window size, *w* is the window data, *ot* denotes “on the threshold” and *ut* denotes “under the threshold”, *u* (the trend in the noise data) is defined as follows:

(3) $$\begin{array}{ll} u & =ot,if\;w_{s}=ot\;\text{and}\;w_{k}=ot\\ & =ut,\text{else}\;if\;w_{s}=ut\;\text{and}\;w_{k}=ut \end{array}$$

If the data on both sides of window are on the threshold, the trend is *ot*, Otherwise, it is *ut*. Third, assuming that *NoU* is the number of data points, the filtering method calculates the calibration data according to the following equation:

(4) $$C_{i}=\frac{1}{NoU}\sum_{n=s}^{k}u_{n}$$

The value of *C_i_* is calculated for the window defined by *s* < *i* < *k*.

The data that do not match the trends in the window are corrected to the *C_i_*, as shown in Figure 4a-2,b-2. Using this filtering method, noise data are removed and correct data are detected, as shown in Figure 4a-3,b-3.

In addition, only one type of noise is filtered out by three window size of mask, but there are many types of noise collected in two or more groups of vehicle data. In this study, to determine the best way to perform noise filtering, experiments were conducted using the proposed system and three to nine window size.

##### 3.2.3. Detection of Vehicles and Vehicle Positions

**Figure 5.**
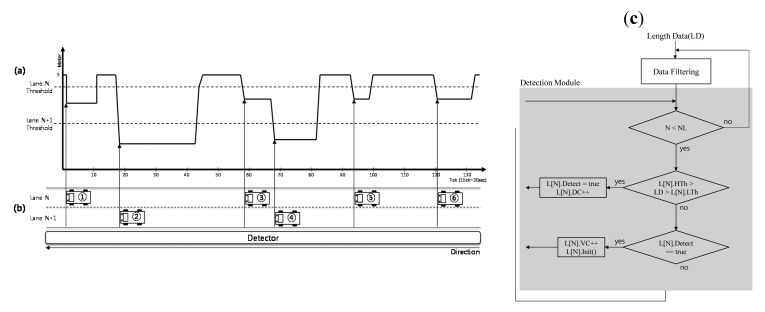
(**a**) Detected data from actual road, (**b**) location of vehicle on the road and (**c**) vehicle detection algorithm.

Figure 5 represents the filtered vehicle data received as vehicles pass through the range of the sensor in each lane. Using the thresholds shown in Figure 5a, the proposed system can detect vehicles in the lanes and determine their locations.

The presence of vehicles is determined according to the threshold of the lane. For example, if Vehicle 1 in Lane N enters the range of the sensor, data are received from the carriers of the ultrasonic sensor. The values are located between the thresholds of Lane N and Lane N+1. The horizontal lines in Figure 5a represent the range of these thresholds. While Vehicle 1 is within the range of the sensor, its data are also within the range of the Lane N threshold. After a few seconds, when the vehicle leaves the range of the sensor, the data passes over the threshold of Lane N. As a result, one vehicle is detected. Additionally, there are different patterns for detecting vehicles. Because Vehicle 3 passes under the threshold of Lane N, Vehicle 4 immediately passes under the threshold of Lane N+1. In this situation, Vehicle 3 is detected by the threshold of Lane N, and Vehicle 4 is detected by the threshold of Lane N+1.

Second, the location of each vehicle can be determined from the value of the threshold for each lane. Because they are different values, the lanes can be distinguished. For example, when a vehicle enters a sensor zone, if the sensing data are between the value of Lane N's threshold and the value of Lane N's threshold, we can determine that this vehicle is located in Lane N. Also, when there are additional lanes in the road, we can determine the vehicle location in the cases of more than Lane N according to threshold.

When there are additional lanes in the road, the proposed system can determine the vehicle location in the cases of more than Lane N because we define the thresholds that represent the minimum boundary values for establishing the presence of vehicle in each lane. The threshold boundaries are calculated with reference to widths of lanes. Eventually, the sensing distance of ultrasonic is equal to the threshold boundaries or width of lanes, and can be adjusted by number of lane. Thus, proposed system can separate the vehicle and noise as using calculated threshold in distances bigger than Lane N or more, and there will not be interference between vehicles going in opposite directions.

Additionally, When there is congested traffic on the road and vehicles in adjacent lanes do not appear to have any gaps, sensors could only detect one the vehicle, closer to the sensor and has missing errors. To address these types of problems, an additional sensors could be installed on the other side of the road. If sensors are on both sides of a road, the lanes are divided by 2, and each divided lane is included in one of both sensors. Sensing distance of each sensor is adjusted by number of lanes which is included in sensor. Also, when the sensors are installed on both sides of a road that contains the odd-lanes as a three-lanes or more, one of both sensor should contain the remaining one-lane after divided by 2.

Figure 5c shows a flow chart of the vehicle detection algorithm described above. First, the data are filtered by the “Data Filtering” module to remove noise. Second, in the detection module, the processing is repeated according to number of lanes (NL). For example, for a two-lane road, if the vehicle enters under the threshold of Lane 1 (L[1].HTh > LD > L[1].LTh, HTh: High Threshold, LTh: Low Threshold), the detection mode (L[1].Detect) of the current data is set to “true.” If the vehicle escapes from the threshold of Lane 1, the vehicle count of Lane 1, (L[1].VC), is incremented by 1. “L[1].Detect” is re-set to “false” again to prepare for the next detection processing cycle. After the processing for Lane 1 is finished, this algorithm executes the detection process for the vehicle in Lane 2. The data structure for Lane 2 (L[2]) is used in the next loop of processing.

#### 3.3. Wireless Ultrasonic Sensor Mote (WUSM)

**Figure 6.**
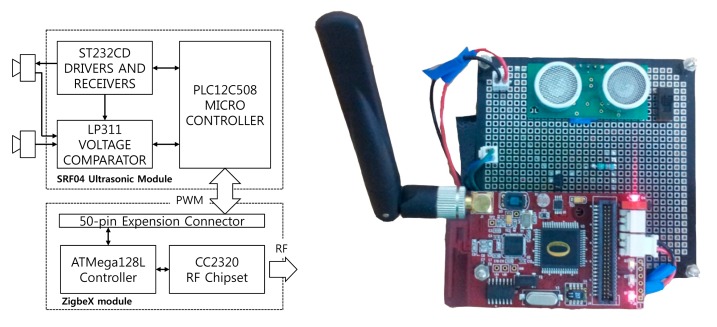
Vehicle detector hardware design.

In this study, we developed an ultrasonic sensor module that is easy to establish and maintain and is highly mobile, because of the miniaturization of the module. Figure 6 shows the structure of the ultrasonic sensor and communication module. Based on the architecture of the model, we developed the wireless ultrasonic sensor node.

The sensor module was developed with a Devantech SRF-04 ultrasonic sensor and its maximum detection range is up to 7 m, which is greater than that of other sensors. [7] The angle of the ultrasonic transmitter is 90 degree used on SRF series. Actually, the sensor received the reflected pulses in 40–45 degrees. An ultrasonic sensor with a range greater than that of the sensor used in this study could detect vehicles in additional lanes farther away from the sensor because the maximum detection range depends on the capabilities of the ultrasonic sensors made by different companies. Also sampling rate is determined to 20 Hz. The interval of ultrasonic bursts can be determined from minimum value 10 ms to maximum value 200 ms. Since the detection interval is determined to max value 200 ms, it can reduce a lot of power consumption of the sensor, but may not be able to detect a vehicle because speed of the vehicle is vary while the ultrasonic burst does not occur from sensor. Furthermore, the fast interval of bursts can detect the most of vehicle but increases the power consumption. Thus, we priority decide that the interval of ultrasonic bursts is 50 ms which is fast rate and reduces the power consumption. 50 ms is converted to 20 Hz sampling rate. The WUSM is manufactured without using battery power. However the ultimate goal of our system is the vehicle detecting system based on Wireless Sensor Network without restriction of the location. Thus, future work, we should research a new method such as sampling rate or sensing distance to consider the battery consumption of WUSM. The SRF-04 ultrasonic sensor in the proposed system consist of ST232CD drivers and receivers. The main module consists of a ZigBeX based ATMega128L controller and CC2320 radio transceiver [13].

### 4. Experiments and Results

#### 4.1. Experimental Environment

**Figure 7.**
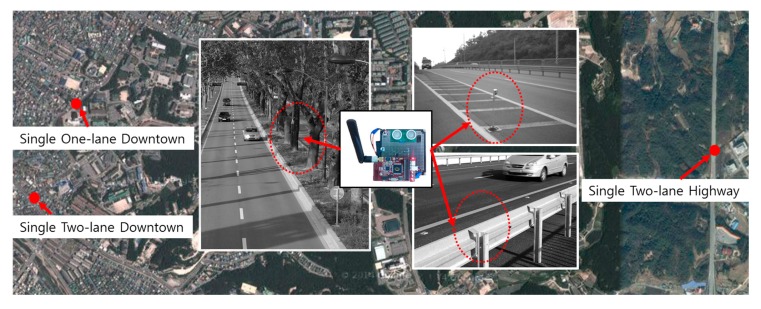
Installed sensor on two-lane road, downtown (left) and highway (right).

Figure 7 notes that the sensors are tested in the three place (red point) for experiments and the cases that the sensors are installed to various structure (the center of the figure). First of all, the system was installed and tested on a single one-lane road, a single two-lane downtown road, and a single two-lane highway in the city of Chuncheon in the province of Kangwon in the Republic of Korea.

The single two-lane downtown road has a speed limit of 70 km/h. It experiences heavy traffic flow and congestion in the daylight. The single one-lane downtown road has a speed limit of 60 km/h and is congested in the morning and evening. These sections were used to compare the accuracy of the system for the same traffic flow. The single two-lane highway has a speed limit of 80 km/h. This section was used to assess the accuracy of the proposed system in detecting vehicles passing through the sensor zone at high speed, in comparison to its accuracy in detecting vehicles on the two-lane downtown road. To detect vehicles on the road, ultrasonic sensors were installed on the side of the roads. There are various installation methods for our system. The structures installed on the side of the road (e.g., street trees, guardrails or streetlamp *etc.*) can be used as an installation place of WUSM as shown in the center of the Figure 7. Also, they can be fabricated and installed directly on the side of the road. The primary criterion considered in the performance evaluation was the accuracy of detection of the traffic flow and the location of vehicles in the multiple lanes. Tests were conducted during the daytime and nighttime.

#### 4.2. Vehicle Detection Results

A WUSM installed on the side of a road can detect up to two lanes of traffic using one device. Therefore, the experiments had to be planned according to the number of lanes and the amount of traffic at various locations. To compare the accuracy of traffic volume determination in real environments, we measured the traffic volumes on individual one-lane and two-lane roads. In addition, tests were conducted on individual two-lane roads with different levels of traffic flow to assess the accuracy of the system in detecting and locating vehicles in the lanes.

Table 1 shows the data on the actual traffic flow and detected traffic flow on the single two-lane road. There is heavy traffic flow and congestion at rush hour and the error rates were 3.0% during daytime testing and 2.8% during nighttime testing. Note that there were 15 missing data items for the daytime testing, shown as missing data in the table. When there was heavy traffic on the road, if the vehicles in Lane 1 and Lane 2 passed through the range of the WUSM, which was installed on the side of the road, at the same time, the sensor could only detect the vehicle in Lane 2, closer to the sensor. In addition, if a vehicle changed lanes within the range of the WUSM, its location could not be detected, which resulted in missing data and overcounting errors. Also if WUSM is obstructed by parked vehicles, it cannot detect any vehicles on the road. Therefore, any vehicles should not be parked within the range of the sensor.

Table 2 show the data for the actual and detected traffic flow on the single one-lane road in the downtown areas. These results can be compared with the results shown in Table 1. This road is a single one-lane road, but it is as heavily trafficked and congested as the single two-lane road in the downtown area (Table 1) because many people use this road to go to the downtown area or the university. The data for the single one-lane road do not exhibit errors due to vehicles changing lanes or vehicles overlapping, as the data for the single two-lane road show. However, if vehicles stay in the sensor's range for long periods of time because of congestion, error due to noise data can result.

The detection results for the single two-lane highway with low traffic flow are shown in Table 3. This road is in the outer portion of the city and is lightly trafficked. The traffic volume at night is 50% lower than in the daytime. We know that the WUSM experienced frequent errors in monitoring the congested traffic flow on the single two-lane downtown road. It has been noted that the WUSM produced relatively high error, *i.e*., 3% false rate, in detecting the vehicles in a congested flow. To address these types of problems and reduce the error rate, an additional WUSM could be installed on the other side of the road. The results for the single one-lane road downtown indicate relatively accurate detection of 0.3% and 0.7%, and the results for the two-lane highway indicate a low error rate because that highway has less traffic than the single two-lane downtown road.

#### 4.3. Noise Filtering Results According to Window Mask Size

Noise data usually are generated as a vehicle approaches the sensor's angle or departs from it. When the vehicle departs from the sensor's angle, the sensor receives a small amount of noise because the surface area of the vehicle reflected by the carrier is also small. Therefore, the noise of various sizes can be generated according to characteristics of the vehicles in the measurement process and cannot be simply removed using a fixed filter window size. To find optimal filter size from the noise of various sizes, we experimented the error rates of the proposed system for filter window sizes of 3–9 cells. The experimental results can be used to determine the best filter size to most effectively eliminate noise by the vehicle detection system.

**Figure 8.**
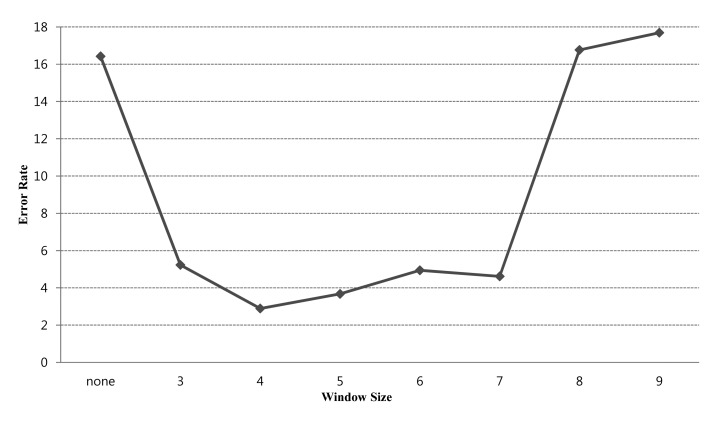
Overall error rate according to filter window size.

Figure 8 shows the error rate results according to the window size of the filtering mask. Each error rate is the average value of detection errors measured for each window size. The data measured in daytime and nighttime on three types of roads were used in the analysis. The results show that if a noise filter is not used, the error rate may be up to 16% However, if a noise filter is applied, the error rate decreases by 3%–6%, depending on the filter window size. The results show that the lowest value of 2.9% corresponds to a window size of 4 and that if the window size exceeds 4 the error rate increases.

### 5. Conclusions

Traffic surveillance technologies can be classified as intrusive or non-intrusive technologies. Intrusive traffic sensors are installed within or across a pavement and have drawbacks that include high maintenance costs and disruption of traffic for installation, repair, or system reinstallation for road repairs or resurfacing. Non-intrusive sensors can be installed above or on the side of roads with minimum disruption to traffic flow and therefore offer the advantages of low installation costs and ease of maintenance. However, one of the disadvantages of non-intrusive sensors is that one must be installed for each lane.

In this paper, we propose a system for measuring vehicles using ultrasonic sensors mounted at the side of the road. The proposed algorithm consists of three parts can accurately detect vehicles in multiple lanes.

The proposed method was found to exhibit error rates of up to 3%, compared with actual traffic flow measurements, at three locations under two conditions (daytime and nighttime). However, there were very few differences in the results for a single two-lane downtown road and a rural highway road. Missing-vehicle errors occurred when multiple vehicles entered the sensor zone simultaneously. This happened more often on the heavily congested road. However, its effect was minimal in our experimental results.

The proposed ultrasonic sensor is small and can communicate with other sensors through a wireless network. Therefore, if sensors are installed on both sides of a road, vehicles can be detected more accurately than if a sensor is installed on only one side. In addition, because the WUSM is low in cost and easy to maintain, it can be used on country roads as well as complex metropolitan roads. Because of the low error rates achieved in vehicle detection and its low installation cost, the WUSM can contribute to vehicle detection as part of an ITS.

In future work, we plan to apply the sensor network system to a more extensive traffic environment. Therefore, the power management of our system will be considered so that the sensors can be maintained for a long time and remain able to transfer data.

## Figures and Tables

**Table 1.** Detection result for single two-lane road downtown.

**Time**	**Actual Passing Vehicle (Vehicles)**			**Detected Vehicle (Vehicles)**			**Error Rate (Percent)**			**Type of Error (Vehicles)**					

										**Missing**			**Overcounting**		

	**Lane 1**	**Lane 2**	**Total**	**Lane 1**	**Lane 2**	**Total**	**Lane 1**	**Lane 2**	**Total**	**Lane 1**	**Lane 2**	**Total**	**Lane 1**	**Lane 2**	**Total**
11:00–11:10	144	24	168	142	26	168	2.8	8.3	3.6	3	0	3	1	2	3
11:10–11:20	133	40	173	128	42	170	3.8	5.0	4.0	5	0	5	0	2	2
11:20–11:30	108	22	130	106	24	130	1.9	9.1	3.1	2	0	2	0	2	2
11:30–11:40	134	33	167	133	34	167	2.2	3.0	2.4	2	0	2	1	1	2
11:40–11:50	139	56	195	137	56	193	2.9	0.0	2.1	3	0	3	1	0	1

Total	658	175	833	646	182	828	2.7	5.1	3.0	15	0	15	3	7	10

20:00–20:10	114	37	151	115	37	152	2.6	0.0	2.0	1	0	1	2	0	2
20:10–20:20	121	41	162	124	41	165	4.1	0.0	3.1	1	0	1	4	0	4
20:20–20:30	112	31	143	114	33	147	1.8	12.9	4.2	0	1	1	2	3	5
20:30–20:40	148	32	180	148	30	178	0.0	12.5	2.2	0	3	3	0	1	1
20:40–20.50	157	21	178	157	22	179	2.5	4.8	2.8	2	0	2	2	1	3

Total	652	162	814	658	163	821	2.2	6.0	2.8	4	4	8	10	5	15

**Table 2.** Detection result for single lanes downtown.

**Time**	**Actual Passing Vehicle (Vehicles)**			**Detected Vehicle (Vehicles)**			**Error Rate (Percent)**			**Type of Error (Vehicles)**					

										**Missing**			**Overcounting**		

	**Lane 1**	**Lane 2**	**Total**	**Lane 1**	**Lane 2**	**Total**	**Lane 1**	**Lane 2**	**Total**	**Lane 1**	**Lane 2**	**Total**	**Lane 1**	**Lane 2**	**Total**
11:00–11:10	78	0	78	78	0	78	0	0	0	0	0	0	0	0	0
11:10–11:20	76	0	76	76	0	76	0	0	0	0	0	0	0	0	0
11:20–11:30	68	0	68	68	0	68	0	0	0	0	0	0	0	0	0
11:30–11:40	73	0	73	73	0	73	0	0	0	0	0	0	0	0	0
11:40–11:50	83	0	83	84	0	84	1.2	0	1.2	0	0	0	1	0	1

Total	378	0	378	379	0	379	0.3	0	0.3	0	0	0	1	0	1

20:00–20:10	77	0	77	78	0	78	1.3	0	1.3	0	0	0	1	0	1
20:10–20:20	86	0	86	86	0	86	0	0	0	0	0	0	0	0	0
20:20–20:30	80	0	80	80	0	80	0	0	0	0	0	0	0	0	0
20:30–20:40	91	0	91	92	0	92	1.1	0	1.1	0	0	0	1	0	1
20:40–20:50	85	0	85	86	0	86	1.2	0	1.2	0	0	0	1	0	1

Total	419	0	419	422	0	422	0.7	0.0	0.7	0	0	0	3	0	3

**Table 3.** Detection result for single two-lane highway.

**Time**	**Actual Passing Vehicle (Vehicles)**			**Detected Vehicle (Vehicles)**			**Error Rate (Percent)**			**Type of Error (Vehicles)**					

										**Missing**			**Overcounting**		

	**Lane 1**	**Lane 2**	**Total**	**Lane 1**	**Lane 2**	**Total**	**Lane 1**	**Lane 2**	**Total**	**Lane 1**	**Lane 2**	**Total**	**Lane 1**	**Lane 2**	**Total**
11:00–11:10	38	56	94	35	56	91	7.89	0	3.1	3	0	3	0	0	0
11:10–11:20	61	54	115	61	55	116	0	1.8	0.8	0	0	0	0	1	1
11:20–11:30	71	46	117	68	48	116	4.2	4.3	4.2	3	0	3	0	2	2
11:30–11:40	53	44	97	53	47	100	0	6.8	3.0	0	0	0	0	3	3
11:40–11:50	44	38	82	42	38	80	4.5	0	2.4	2	0	2	0	0	0

Total	267	238	505	259	244	503	3.0	2.5	2.8	8	0	8	0	6	6

20:00–20:10	26	16	42	25	16	41	3.8	0	2.3	1	0	1	0	0	0
20:10–20:20	32	16	48	32	17	49	0	6.2	2.0	0	0	0	0	1	1
20:20–20:30	27	16	43	27	16	43	0	0	0	0	0	0	0	2	2
20:30–20:40	27	13	40	27	14	41	3.7	0	2.5	0	0	0	1	0	1
20:40–20:50	21	13	34	22	13	35	4.7	0	2.9	0	0	0	1	0	1

Total	133	74	207	133	76	209	2.3	1.4	1.9	1	0	1	2	3	5
